# The InterPro protein families database: the classification resource after 15 years

**DOI:** 10.1093/nar/gku1243

**Published:** 2014-11-26

**Authors:** Alex Mitchell, Hsin-Yu Chang, Louise Daugherty, Matthew Fraser, Sarah Hunter, Rodrigo Lopez, Craig McAnulla, Conor McMenamin, Gift Nuka, Sebastien Pesseat, Amaia Sangrador-Vegas, Maxim Scheremetjew, Claudia Rato, Siew-Yit Yong, Alex Bateman, Marco Punta, Teresa K. Attwood, Christian J.A. Sigrist, Nicole Redaschi, Catherine Rivoire, Ioannis Xenarios, Daniel Kahn, Dominique Guyot, Peer Bork, Ivica Letunic, Julian Gough, Matt Oates, Daniel Haft, Hongzhan Huang, Darren A. Natale, Cathy H. Wu, Christine Orengo, Ian Sillitoe, Huaiyu Mi, Paul D. Thomas, Robert D. Finn

**Affiliations:** 1 European Molecular Biology Laboratory, European Bioinformatics Institute (EMBL-EBI), Wellcome Trust Genome Campus, Hinxton, Cambridge, CB10 1SD, UK; 2 Faculty of Life Science and School of Computer Science, The University of Manchester, Manchester, M13 9PL, UK; 3 Swiss Institute of Bioinformatics (SIB), CMU - Rue Michel-Servet, 1211 Geneva 4, Switzerland; 4 Center for Integrative Genomics, University of Lausanne, 1015 Lausanne, Switzerland; 5 Department of Biochemistry, University of Geneva, 1211 Geneva, Switzerland; 6 Pôle Rhône-Alpin de Bio-Informatique (PRABI), Batiment G. Mendel, Universite Claude Bernard, 43 bd du 11 novembre 1918, 69622 Villeurbanne Cedex, France; 7 European Molecular Laboratory (EMBL), Meyerhofstasse 1, 69117 Heidelberg, Germany; 8 Department of Computer Science, University of Bristol, Woodland Road, Bristol, BS8 1UB, UK; 9 J. Craig Venter Institute (JCVI), 9704 Medical Center Drive, Rockville, MD 20850, USA; 10 Protein Information Resource (PIR), Georgetown University Medical Center, Washington, DC 20007, USA; 11 Center for Bioinformatics and Computational Biology, University of Delaware, Newark, DE 19711, USA; 12 Structural and Molecular Biology Department, University College London, University of London, London, WC1E 6BT, UK; 13 Division of Bioinformatics, Department of Preventive Medicine, University of Southern California, Los Angeles, CA 90089, USA

## Abstract

The InterPro database (http://www.ebi.ac.uk/interpro/) is a freely available resource that can be used to classify sequences into protein families and to predict the presence of important domains and sites. Central to the InterPro database are predictive models, known as signatures, from a range of different protein family databases that have different biological focuses and use different methodological approaches to classify protein families and domains. InterPro integrates these signatures, capitalizing on the respective strengths of the individual databases, to produce a powerful protein classification resource. Here, we report on the status of InterPro as it enters its 15th year of operation, and give an overview of new developments with the database and its associated Web interfaces and software. In particular, the new domain architecture search tool is described and the process of mapping of Gene Ontology terms to InterPro is outlined. We also discuss the challenges faced by the resource given the explosive growth in sequence data in recent years. InterPro (version 48.0) contains 36 766 member database signatures integrated into 26 238 InterPro entries, an increase of over 3993 entries (5081 signatures), since 2012.

## INTRODUCTION

InterPro was originally launched in beta in October 1999, with a full version 1.0 release in March the following year. From an initial core of four source databases (Pfam (1), PRINTS (2), PROSITE (3) and ProDom (4)), InterPro has expanded, so that it now integrates signatures from seven additional repositories: CATH-Gene3D (5), HAMAP (6), PANTHER (7), PIRSF (8), SMART (9), SUPERFAMILY (10) and TIGRFAMs (11). Each source database has its own distinct biological focus and/or method of signature production. The aim of InterPro is to combine their individual strengths to provide a single resource, through which scientists can access comprehensive information about protein families, domains and functional sites. The InterPro database does not generate diagnostic models itself, but rather, groups one or more related member database signatures, and provides additional overarching functional annotations, including Gene Ontology (GO) (12) terms wherever possible. Once a member database signature is categorized by InterPro, that database signature is considered ‘integrated’.

### Member database integrations

In more detail, InterPro entries are typically constructed as follows:

*Signature generation*: Protein family databases from the InterPro consortium identify groups of homologous protein sequences, based on sequence similarity and function. They use these sets of sequences to construct representative signatures that are used to iteratively search large sequence databases, such as the UniProt Knowledgebase (UniProtKB) (13), until no more proteins can be classified into the group. Upon member database release, these signatures are passed to InterPro for integration.

**Figure 1.**
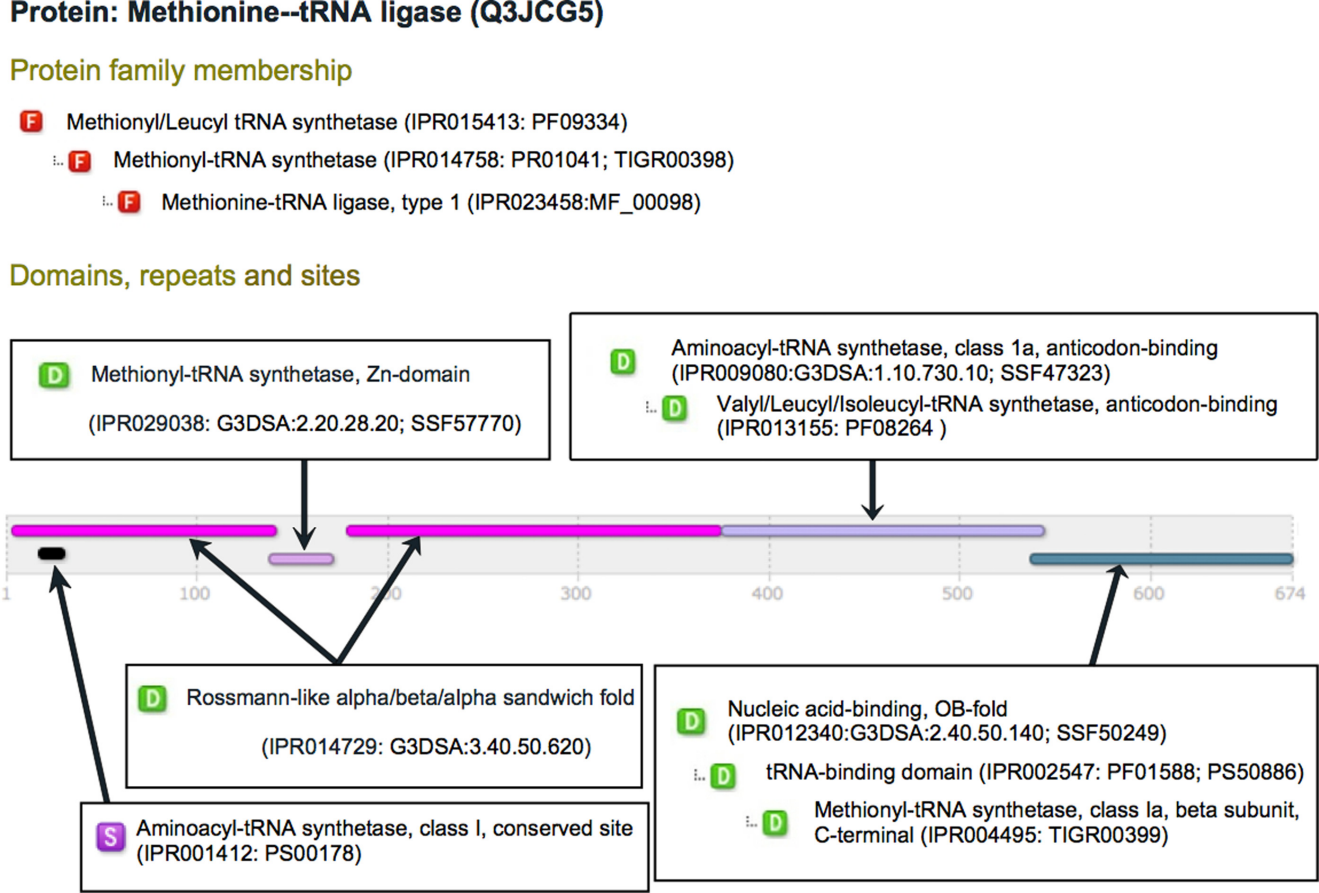
InterPro matches for UniProtKB entry Q3JCG5 showing predicted protein family membership, domains and sites.

*Signature integration*: At InterPro, the matches between the latest version of UniProtKB and the new signatures (and all other signatures) are determined and manually inspected by curators to ensure that they are accurate. Aberrant signatures, both old and new, that generate false positive matches are identified and reported back to the member databases. The new signatures passing quality control are added to InterPro. Each InterPro entry is annotated with a name, a descriptive abstract and GO terms. Hierarchical relationships are identified between the entries, tracing those entries that represent smaller, functionally specific subfamilies of larger families, or specific subclasses of broader classes of domain (see Figure 1). Semi-automatic procedures create and maintain links to a range of other databases, including the protein interaction database IntAct (14), the ENZYME (15), MetaCyc (16), UniPathway (17) and KEGG (18) databases, and the 3D structure database PDB (19).

InterPro signature matches to UniProtKB and to the UniParc protein sequence archive are regularly calculated using the InterProScan software package (20). This information is made available to the public via XML files and the database's Web interfaces, which users can search with either a protein sequence or a protein identifier. The InterPro matches are also used to aid UniProtKB curators in their annotation of Swiss-Prot proteins, and are utilized by the automated systems that add annotation to UniProtKB/TrEMBL.

Figure 1 summarizes the InterPro matches for UniProtKB entry Q3JCG5, a methionine–tRNA ligase from *Nitrosococcus oceani*, highlighting the value of the signature-integration process. According to a number of hierarchically-related InterPro entries, the protein can be classified into the methionine–tRNA ligase family (specifically, the type 1 subclass of this family), which is part of the wider family of methionyl/leucyl tRNA synthetases. InterPro also predicts the protein to have a Rossmann fold, the two halves of which (shown in InterPro as two domains) are linked by a zinc-binding connective peptide domain. Two further domains are predicted—an anti-codon binding domain and a C-terminal domain. InterPro identifies these domains as subclasses of broader classes of domain, as indicated in the domain hierarchy annotation (for example, the C-terminal domain is predicted to be a specialized type of nucleic acid binding domain). Finally, towards the N-terminus of the protein, InterPro identifies a conserved site that is specific to class I aminoacyl–tRNA synthetases.

**Figure 2.**
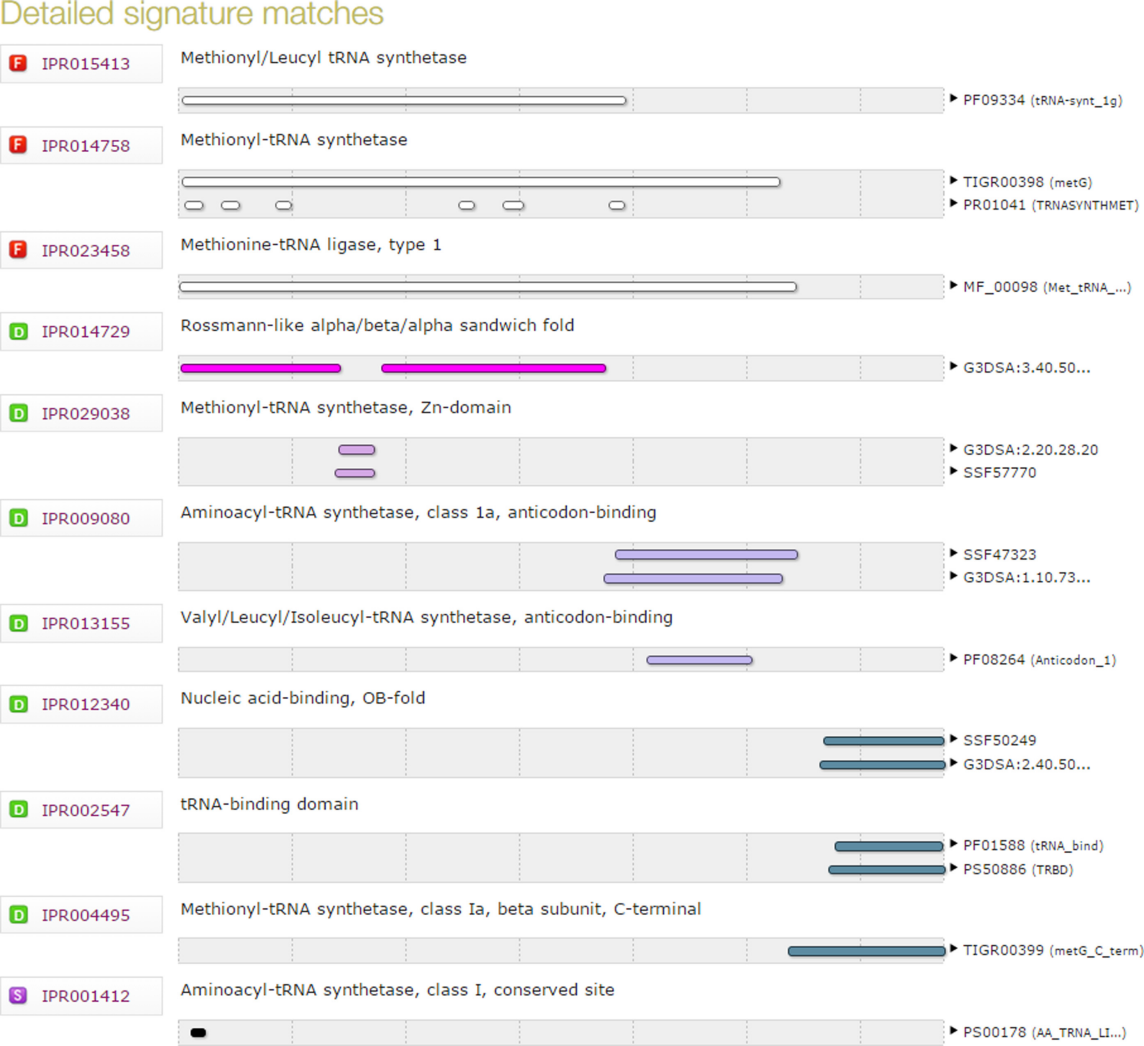
Detailed InterPro member database match data for UniProtKB entry Q3JCG5.

The data used to generate the summary were drawn from 16 individual signatures from seven member databases, as illustrated in Figure 2. By integrating signatures that represent the same biological entity into 11 entries, InterPro reduces redundancy. By tracing the relationships between the entries, InterPro helps rationalize the protein match data and aids interpretation and summarization of the results. It is worth noting that the average number of member signatures per InterPro entry is 1.4, the highest number of signatures in an entry being eight. The average number of signatures per entry is diminishing over time, as the member databases have the large protein families adequately represented and are now generating new signatures that cover (smaller) areas of protein space that are rarely represented in other databases. Nevertheless, there remain cases where new protein families will still be represented by multiple databases; for example, a publication describing a novel structure is likely to lead to that protein family being represented in CATH-Gene3D, Pfam and/or SUPERFAMILY.

### Growth and coverage of the database

New database entries continue to be integrated into InterPro, and there have been 14 public releases since the last update (release 34.0 in 2012 (21)), with an additional 5081 signatures being integrated into 3993 new entries. The latest release (version 48.0) contains 36 766 member database signatures integrated into 26 238 InterPro entries, and provides matches to 83.5% of the sequences in UniProtKB release 2014_07 (see Table 1), an increase of 3.9% compared with release 34.0.

**Table 1.** Coverage of the major sequence databases UniProtKB and UniParc (the non-redundant protein sequence archive) by InterPro signatures

Sequence database	Number of proteins in database	Number of proteins with one or more matches to InterPro
UniProtKB/Swiss-Prot	546 000	525 376 (96.2%)
UniProtKB/TrEMBL	79 824 243	66 591 418 (83.4%)
UniProtKB (total)	80 370 243	67 116 794 (83.5%)
UniParc	67 862 204	55 078 104 (81.2%)

**Figure 3.**
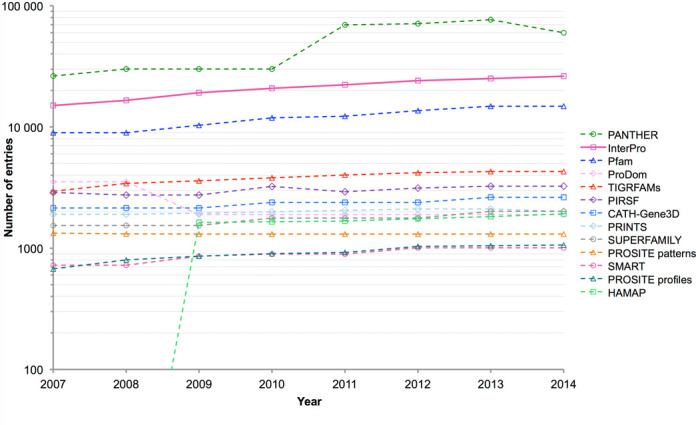
Number of entries provided by InterPro and its member databases per year.

These increases are set against a constant background of flux in InterPro's member databases. The number of entries provided by each resource since 2007 (when InterPro began collating these data) is shown in Figure 3, along with the growth in InterPro entries over the same time period. Most of the databases show a steady rate of increase over time, while some, such as ProDom and PANTHER, fluctuated in size.

The percentage of entries integrated into InterPro for each member database is shown in Table 2. As can be seen, the level of integrated entries is in excess of 90% for all databases, except CATH-Gene3D, Superfamily, PANTHER and ProDom. The former two both define domains based on known 3D structures and tend to represent the broadest entries in InterPro. However, as they are based on two different structural classification resources (CATH (22) and SCOP (23), respectively), there can be differences in scope and domain boundaries, which can make integration problematic. However, the two databases have been working towards greater alignment through the Genome3D project (24), which should help address this issue. The current release of PANTHER, meanwhile, provides almost 60 000 signatures, far in excess of any other member database, resulting in a considerable backlog of entries to work through. In addition, two PANTHER releases involved significant changes (see Figure 3): in 2010 a new phylogenetic tree reconstruction algorithm and reference sequence set were introduced (25), and in 2013 subfamily boundaries within families were refined to increase consistency. Any entries removed from PANTHER during the rebuilding process are also deleted from InterPro, so that the integration of PANTHER entries into InterPro is not an entirely cumulative process. Finally, ProDom is an automatically generated clustering of sequence space, so the regions provided by ProDom do not always reflect protein family domain boundaries; they also lack annotation. Thus, ProDom entries are only integrated when there is correspondence with another signature. However, the presence of an un-integrated ProDom entry on a protein can indicate the presence of a conserved region that may form a functional domain that is yet to be modelled by any other member database.

**Table 2.** Release version and number of member database signatures integrated into InterPro release 48.0

Database	Release number	Total signatures	Integrated signatures	Integrated signatures (%)
CATH-Gene3D	3.5.0	2626	1718	65.4
HAMAP	201311.27	1916	1912	99.8
PANTHER	9.0	59 948	3673	6.1
PIRSF	2.84	3251	3225	99.2
PRINTS	42	2106	2024	96.1
PROSITE patterns	20.97	1308	1290	98.6
PROSITE profiles	20.97	1062	1038	97.7
Pfam	27	14 831	14 134	95.3
ProDom	2006.1	1894	1117	59.0
SMART	6.2	1008	998	99.0
SUPERFAMILY	1.75	2019	1372	68.0
TIGRFAMs	13	4284	4265	99.6

### Mapping to GO terms

The GO provides a controlled vocabulary that can be used to describe gene products in terms of their molecular functions, biological processes and the subcellular location in which they are found, in a consistent and structured fashion. InterPro entries are manually annotated with these terms, allowing GO terms to be inferred for sequences that match the entries, as part of the InterPro2GO pipeline. To date, over 11 500 InterPro entries have been annotated with one or more GO terms, with over 28 000 GO terms in total mapped to the resource. InterPro continues to be a considerable source of GO term annotation: GO terms assigned by InterPro2GO are cross-referenced more than 168 million times in UniProtKB release 2014_07, providing terms for almost 50 million individual proteins.

### Targeted curation of GO terms for bacterial metabolism

Microme (http://www.microme.eu) is a resource for bacterial metabolism that aims to support the large-scale inference of biochemical pathways directly from genome sequence. To this end, Microme has developed a Genome-Reaction Matrix (GRM) containing inferred reactions from thousands of genomes that can be used to build draft metabolic networks and models. Several sources are used for the reaction sets, including InterPro annotations and GO terms, and curated associations of GO functions to reactions in the RhEA database (26). In order to improve the accuracy and coverage of the inference methodology used in the GRM, a Microme curator spent 10 weeks working with the InterPro team, manually reviewing almost 4000 InterPro entries relating to transporters and proteins involved in metabolism, by extracting evidence from published experimental data. Approximately 10% of the InterPro entries examined were then associated with new or improved GO terms.

These new annotations were used to increase the coverage of metabolic and transport reactions in Microme. On a data-set of 5423 genome sequences present in Microme release 3.0, the number of gene-reaction associations increased by 9% (to a total of 4 730 692), the number of genome-reaction associations by 9% (to a total of 2 162 546), and the average number of reactions per genome also by 9% (to a total of 399).

The improved annotations were also propagated to UniProtKB, through the InterPro2GO pipeline, resulting in improved annotations for over 2.5 million sequences, as of release 2014_07. As an additional beneficial outcome, 35 new GO terms were created as part of this work, 74 new reactions were curated into RhEA (with the curation of additional chemical entities in the ChEBI database (27) where necessary to support this) and 113 new GO-RhEA mappings were created.

### New website features

#### Sequence search

In response to user feedback, the sequence search facility on the InterPro website has been refined to include an ‘Advanced option’, allowing users to select which member database and/or additional sequence feature prediction algorithm (i.e. Coils, Phobius (28), TMHMM (29), SignalP (30)) to run. This facility is available under the ‘Search’—‘By sequence’ tabs at the top of the InterPro homepage.

#### Domain architecture search interface

A new domain architecture search tool has been developed and made available via the website (http://www.ebi.ac.uk/interpro/search/domain-organisation); this allows the InterPro database to be searched with a particular domain, or set of domains, returning all of the domain architectures and associated proteins that match the query. This makes it easy to rapidly identify all of the different domain combinations, where one type of domain co-occurs with another, or where a particular domain is followed by another domain (e.g. an SH3 domain is found C-terminal to a protein kinase domain, or vice versa), and to list all of the proteins in UniProtKB with a matching domain architecture.

The tool is specifically designed to work with InterPro entries of type *domain*, which represent distinct functional or structural units that may be found in different proteins with a range of overall functions. It is these entries that typically recombine to provide functional diversity and are the subject of combinatorial searches. InterPro entries of type *family* (representing groups of evolutionarily related proteins that share common functions) are not catered for, since such entries tend to be near full length and typically do not undergo recombination.

The tool makes use of a specialized graph-theory-based algorithm that rapidly searches through all domain matches within InterPro and returns proteins that match the domains in the order specified in the query. As InterPro integrates data from a number of different member databases whose domain boundary predictions do not always agree, InterPro domains may overlap. This is in contrast to the ‘beads on a string’ representation that is sometimes used to display domain architectures.

**Figure 4.**
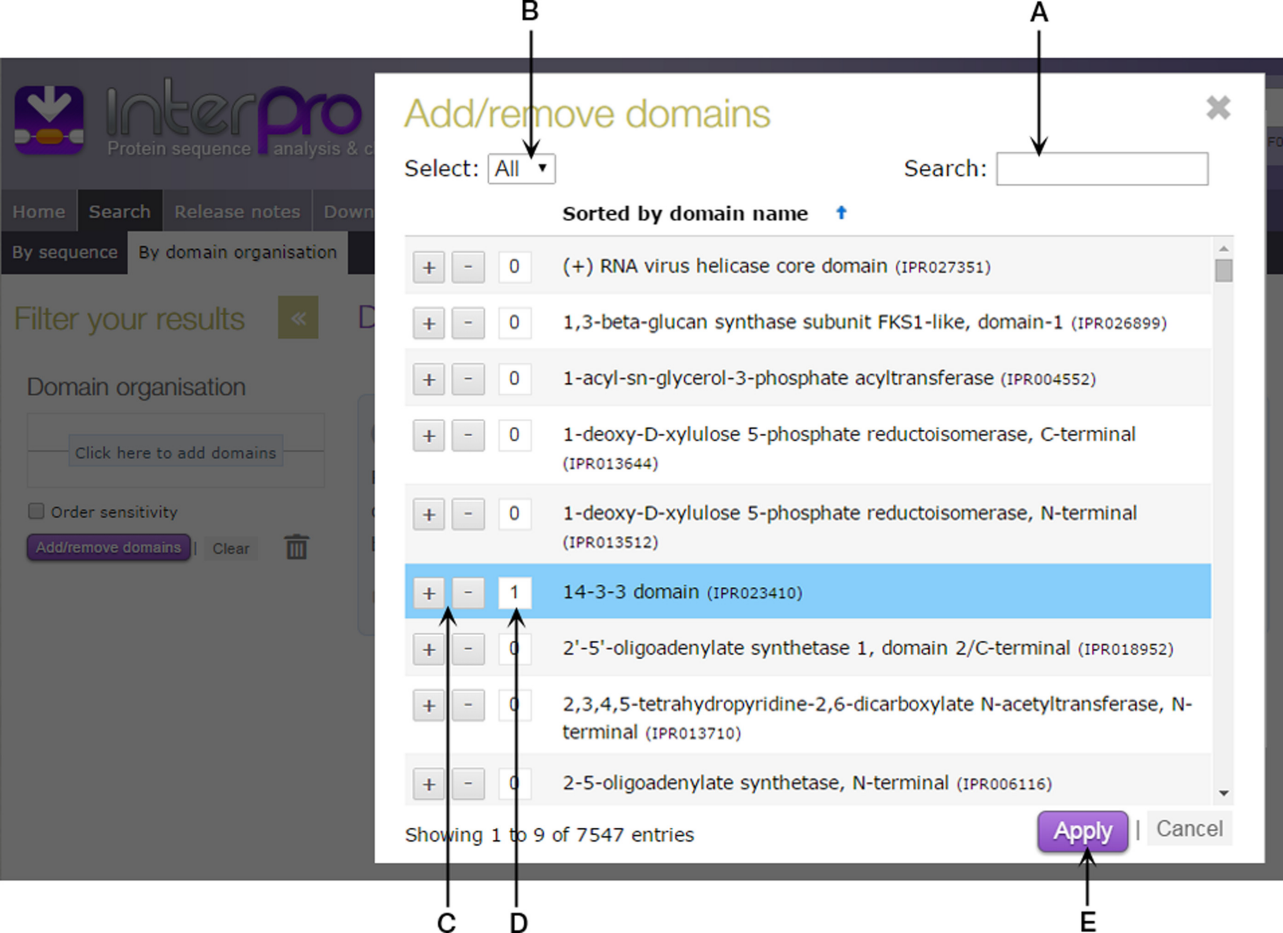
The InterPro Domain Architecture tool add/remove domains pop-up window. The list of domains can be refined using either the search box (**A**) or drop down menu (**B**). Domains can be added or removed from the query using plus or minus buttons (**C**). The number of copies of a particular domain to add to the query is indicated (**D**). Selecting the Apply button (**E**) performs the query.

Users can launch the tool using an interactive panel on the left-hand side of the page. Clicking on this launches a pop-up window with a searchable list of all of the domains in the database (see Figure 4). Once an appropriate domain has been identified from the list, it can be added to or removed from the query using plus and minus buttons next to its name. The same domain can be added to a query multiple times (e.g. to identify all of the proteins in UniProtKB predicted to have two or more pleckstrin homology domains).

**Figure 5.**
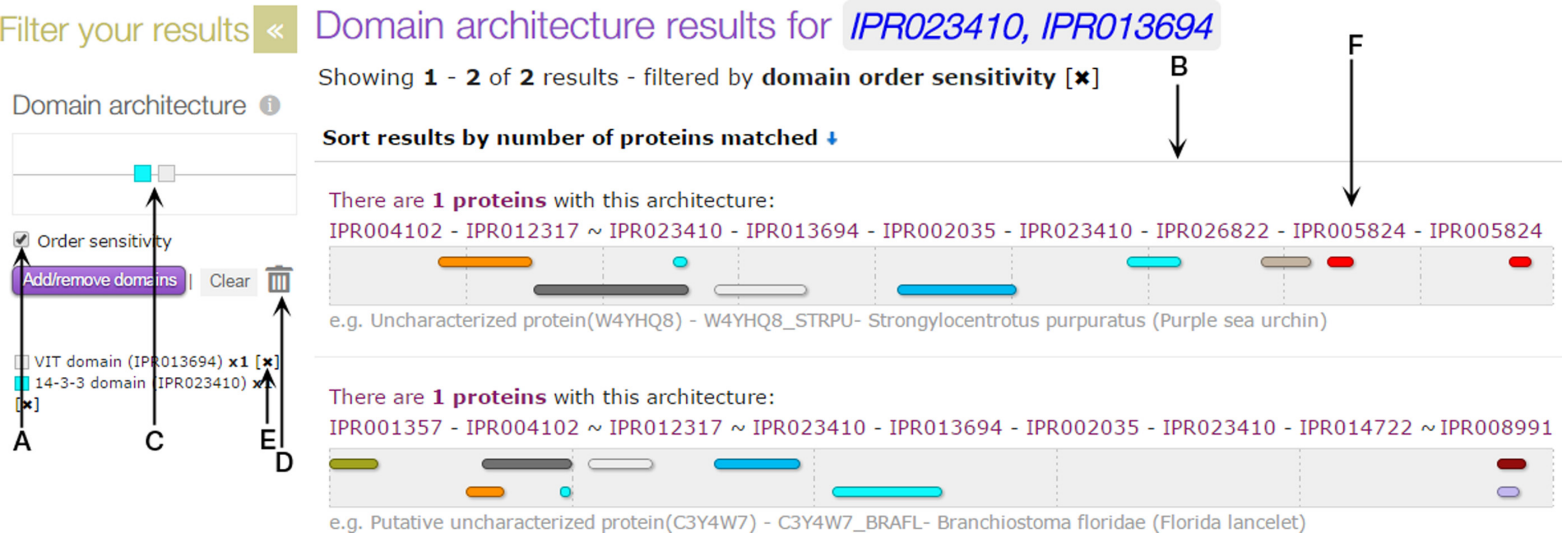
The InterPro Domain Architecture tool showing the results of searching with a VIT and 14-3-3 domain. Checking the ‘Order sensitivity’ option (**A**) means that domain order is taken into account in the results section (**B**). The domains can be reordered by dragging and dropping their graphical representations (**C**), or removed from the query by dragging them to the dustbin (**D**) or clicking on the [x] icon next to their name and accession (**E**). The InterPro accession string (**F**) summarizes the domain architecture composition.

Once the required domains have been selected, pressing the ‘Apply’ button performs the appropriate query. The different domain architectures matching the query, along with the number of proteins matching each domain architecture, are displayed graphically (as shown in Figure 5). A cartoon version of the query is also generated, with domains represented as coloured squares that can be reordered or removed from the query by dragging and dropping, which automatically updates the search results.

## AVAILABILITY

The InterPro database and related software are freely available for download and distribution, provided the appropriate Copyright notice is supplied (as described in the accompanying Release Notes). Data can be downloaded in a flat-file format (XML) and via the Web interfaces described in the text. The InterProScan software is available: (i) as a browser-based tool for analysing single protein sequences (http://www.ebi.ac.uk/interpro/search/sequence-search/); (ii) programmatically via Web services that allow up to 25 sequences to be analysed per request (SOAP-based service documented at http://www.ebi.ac.uk/Tools/webservices/services/pfa/iprscan5_soap and REST-based service at http://www.ebi.ac.uk/Tools/webservices/services/pfa/iprscan5_rest) and (iii) as a downloadable package for local installation (https://code.google.com/p/interproscan/wiki/Introduction).

## DISCUSSION

The rate of growth of sequence data has increased massively in recent years, following the take-up of Next Generation Sequencing (NGS) methodologies. As a consequence, the rate of deposition of nucleotide (and thus protein) sequence data has greatly increased: at the beginning of this century, UniProtKB contained ∼470 000 entries; the current total is ∼80 million, ∼40 million of which were added in the last year alone.

**Figure 6.**
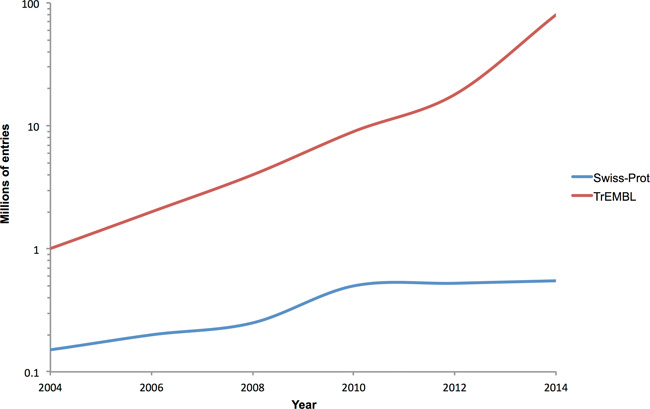
Growth of the manually-annotated Swiss-Prot and automatically annotated TrEMBL sections of UniProtKB over the last decade.

A key challenge facing bioinformatics is the accurate and consistent annotation of these sequences. The scale of this task is illustrated in Figure 6. This graph shows the growth of the manually annotated Swiss-Prot section of UniProtKB versus the exponential increase in UniProtKB/TrEMBL, which has no manual annotation associated with it. Over the last 10 years, UniProtKB's team of dedicated expert Swiss-Prot curators have manually annotated ∼400 000 protein sequences. However, this figure is approximately the number of sequences now entering UniProtKB per *week*, and the rate of sequence deposition is growing.

Clearly, even the most extensive manual efforts cannot meet the challenge of annotating these data. Automated annotation transfer is also beyond the scope of all-against-all pairwise-alignment methods, such as BLAST, due to the sheer scale of the task. Given this, InterPro with its scalable, signature-based approach is more important than ever in providing annotation. The resource has not only managed to keep track with the massive growth of UniProtKB in recent years, but has actually increased its coverage (from 79.6% of proteins in the database in 2011 to 83.5% in 2014). This is owing to a number of factors: the on-going development of new signatures by its partner databases (see Figure 3); the continued integration efforts of InterPro's curators (with only a small percentage of signatures from the majority of member databases awaiting integration—see Table 2); and extensive refactoring by InterPro's developers to ensure that production pipelines continue to scale with the burgeoning data volumes.

While InterPro provides a scalable option in terms of the compression of query terms into signatures, it is important for signature-based protein family databases to constantly review the algorithms and approaches used. The acceleration in search speed using HMMER3 compared with HMMER2 offers a 1000-fold improvement (31), such that a single signature takes, on average, only 12 CPU minutes (using a 2.5 GHz Intel Xeon) to be calculated against 80 million protein sequences. The databases that have adopted HMMER3 do not currently present scalability issues. Other databases, such as HAMAP, hope to achieve similar improvements soon by using the new pfsearchv3 algorithm for their searches, which are currently significantly slower than HMMER3-based methods. Whilst it is advantageous to have diversity between methods, it is important that performance speed is also considered. Based on the projected growth of UniProtKB, it will not be many years before 20 million sequences will be routinely added to each release of the sequence database.

The considerable power of InterPro in propagating annotation is illustrated in the targeted GO curation work. Here, focused curation of a small subset of InterPro entries (∼15% of the database) by an expert curator generated a cumulative ‘snowball’ effect, improving annotation for over 2 million of UniProtKB sequences, with positive knock-on effects on a host of other resources (Microme, RhEA, GO, Reactome and ChEBI). We are very interested in exploring similar collaborations in future, as this approach potentially allows us to rapidly (and with minimal overhead) improve annotation of proteins that may not be adequately covered in UniProtKB at present.

Scalable provision of automatic annotation is important not only for UniProt, but also for other areas of informatics, and improvements in InterPro feed through into other resources. For example, the increased coverage, performance and improved annotation of microbial proteins involved in transport and metabolism will feed into EBI Metagenomics (32), a recently launched Web-based portal for the metagenomics research community, which uses InterProScan to provide functional analysis of metagenomic sequences. We expect that this close association between the two resources will develop further in future (e.g. the pathway prediction functionality developed in InterProScan 5 will be implemented for EBI Metagenomics).
